# Mechanisms of antimicrobial resistance in finfish aquaculture environments

**DOI:** 10.3389/fmicb.2013.00233

**Published:** 2013-08-22

**Authors:** Claudio D. Miranda, Alfredo Tello, Patricia L. Keen

**Affiliations:** ^1^Department of Aquaculture, Universidad Católica del NorteCoquimbo, Chile; ^2^Instituto Tecnológico del Salmón, INTESAL de SalmonChilePuerto Montt, Chile; ^3^Department of Civil Engineering, University of British Columbia, VancouverBC, Canada

**Keywords:** antimicrobials, antimicrobial resistance, fish, aquaculture

## Abstract

Consumer demand for affordable fish drives the ever-growing global aquaculture industry. The intensification and expansion of culture conditions in the production of several finfish species has been coupled with an increase in bacterial fish disease and the need for treatment with antimicrobials. Understanding the molecular mechanisms of antimicrobial resistance prevalent in aquaculture environments is important to design effective disease treatment strategies, to prioritize the use and registration of antimicrobials for aquaculture use, and to assess and minimize potential risks to public health. In this brief article we provide an overview of the molecular mechanisms of antimicrobial resistance in genes found in finfish aquaculture environments and highlight specific research that should provide the basis of sound, science-based policies for the use of antimicrobials in aquaculture.

## INTRODUCTION

Through a continuous process of expansion, intensification, and diversification, aquaculture has become the fastest-growing food industry in the world (Bostock et al., 2010). With production of new species and the expansion of the production of current species to new geographical locations, the risk of disease and need for treatment continues to increase. This risk is compounded by the uncertainties introduced by global climate change, which may affect the emergence and dynamics of new and existing pathogens (Tirado et al., 2010), and consequently the use of antimicrobials and the prevalence of antimicrobial resistance.

The major concern surrounding the use of antimicrobials in aquaculture is considered to be the potential to favor the development of a reservoir of antimicrobial resistance genes (ARGs) that may be eventually transferred to clinically relevant bacteria (FAO/OIE/WHO, 2006). So far, lack of data, methodological constraints, and the complexity of characterizing exposure pathways have prevented the derivation of quantitative estimates of the risk that this may pose to public health. Nevertheless, there is consensus in the scientific community and international organizations concerned with human health, animal health, and food security that steps should be taken to minimize it (FAO/OIE/WHO, 2006; Smith, 2008; Heuer et al., 2009; WHO, 2011). On a global scale, several of the major classes of antimicrobials are being used or have been used in aquaculture. Among these are sulphonamides, penicillins, macrolides, quinolones, phenicols, and tetracyclines (Sapkota et al., 2008), all of which are listed as critically or highly important antimicrobials in human medicine (WHO, 2011). However, the judicious use of antimicrobials in global aquaculture is important to effectively treat bacterial fish diseases and maintain fish health and welfare.

The molecular mechanism, genomic context, and prevalence of genes conferring resistance to antimicrobials determine their clinical relevance (i.e., whether they confer low- vs. high-level clinical resistance, their ability to be mobilized by lateral gene transfer, and their frequency of occurrence). Thus, knowledge of ARGs from aquaculture environments is important to design and prioritize monitoring programs that may generate data that eventually becomes relevant for performing quantitative risk assessments and develop sound treatment strategies to control fish disease. Within this general scope, we provide this article as an overview of the molecular mechanisms, genomic context, and prevalence of quinolone, tetracycline, and phenicol resistance genes that have been reported to occur in aquaculture environments.

## ANTIMICROBIAL RESISTANCE GENES IN AQUACULTURE ENVIRONMENTS

Quinolones (i.e., oxolinic acid, flumequine, and enrofloxacin), tetracyclines [i.e., oxytetracycline (OTC)], and phenicols (i.e., florfenicol) are among the most widely used antimicrobial compounds in aquaculture, and they have been used extensively to control bacterial fish disease in salmon farming [SERNAPESCA (Chile), 2009; Burridge et al., 2010; Rico et al., 2012]. Although the use of quinolones in salmon farming currently accounts for less than 1% of the overall use of antimicrobials, they continue to be used in aquaculture production in several Asian countries [SERNAPESCA (Chile), 2009; Burridge et al., 2010; Rico et al., 2012]. Quinolones, tetracyclines, and phenicols have been reported to be selective for a variety of ARGs and tend to occur in mobile genetic elements that favor their dissemination (i.e., transposons, plasmids, and integrons; Cloeckaert et al., 2000; Chopra and Roberts, 2001; Kümmerer, 2004; Schwarz et al., 2004; Roberts, 2005).

### QUINOLONE RESISTANCE

The protein targets of quinolones are the bacterial enzymes, DNA gyrase and topoisomerase IV. DNA gyrase is a tetrameric enzyme encoded by the *gyrA* and *gyrB* genes, and its main activity is to catalyze the negative supercoiling of bacterial DNA. Topoisomerase IV is also a tetrameric enzyme and it is encoded by the *parC* and *parE* genes; its function is to decatenate and relax the activity of daughter replicons following DNA replication (Anderson et al., 1998; Hawkey, 2003).

The acquisition of quinolone resistance is primarily due to chromosomal mutations in topoisomerases genes (i.e., *gyrA*, *gyrB*, *parC*, and *parE*) and mutations that reduce drug accumulation by decreasing uptake or increasing efflux (Drlica and Zhao, 1997; Ruiz, 2003). Additionally, at least three mechanisms of quinolone resistance are known to be plasmid encoded: (1) Qnr proteins; (2) AAC(6)-Ib-cr aminoglycoside acetyltransferases; and (3) QepA and OqxAB efflux pumps. Qnr proteins protect DNA gyrase and type IV topoisomerase from quinolone inhibition (Tran and Jacoby, 2002; Jacoby, 2005) and the AAC(6)-Ib-cr determinant acetylates several fluoroquinolones. Plasmid-encoded QepA and OqxAB are active efflux pumps that may extrude hydrophilic fluoroquinolones such as enrofloxacin (Li, 2005; Poirel et al., 2008, 2012; Cattoir and Nordmann, 2009; Rodríguez-Martínez et al., 2011).

Various point mutations in the quinolone resistance-determining regions of the *gyrA* and/or *parC* genes have been detected in quinolone-resistant strains of the fish pathogens *Aeromonas hydrophila*, *Vibrio anguillarum*, and *V. parahaemolyticus* (Okuda et al., 1999; Rodkhum et al., 2008; Lukkana et al., 2012). Levels of quinolone resistance in Gram-negative bacteria are suggested to be high when associated with point mutations in both the *gyrA* and *parC* genes, whereas only an intermediate level of resistance is associated with point mutations in the *gyrA* gene only. However, high-level resistance to oxolinic acid associated with a single mutation in the *gyrA* gene has been reported for strains of the fish pathogens *Aeromonas salmonicida*, *Edwardsiella tarda*, and *Photobacterium damselae* (Oppegaard and Sørum, 1994; Goñi-Urriza et al., 2002; Kim et al., 2005, 2011; Ozanne et al., 2005). The extensive administration of quinolones in fish farming has been linked to increased mutations in DNA gyrase and topoisomerase IV in quinolone-resistant fish pathogens such as *Yersinia ruckeri*, *Flavobacterium psychrophilum*, and *V. anguillarum* (Gibello et al., 2004; Izumi and Aranishi, 2004; Colquhoun et al., 2007; Izumi et al., 2007; Shah et al., 2012).

Plasmid-mediated quinolone resistance in bacteria associated with fish farms has been detected in several countries. Ishida et al. (2010) detected the *qnr* and *aac(6′)-Ib-cr* resistance determinants in bacterial strains isolated from fish farm water samples in Egypt. Recently, Buschmann et al. (2012) reported the occurrence of topoisomerase protection genes *qnrA*, *qnrB*, and *qnrS*, the putative enzymatic inactivation gene *aac(69)-Ib-cr* and the efflux pump gene *oqxA* among strains isolated from un-polluted and fish farm-impacted marine sediments in Chile. Jiang et al. (2012) found a high prevalence of *qnrB* and *qnrS* genes in *Escherichia coli* strains recovered from Chinese farmed fish while *qnrD* and *aac(6′)-Ib-cr* genes occurred less frequently. Han et al. (2012) found 17 strains encoding chromosomal mutations in *gyrA*, 11 strains encoding mutations in *parC*, and a few strains carrying the *qnrS1*-like and *qnrS2* genes among 33 *Aeromonas* spp. isolated from diseased fish and from water samples. The identification of QnrS determinants in *Aeromonas* spp. suggests that they may act as an environmental reservoir of *qnrS* genes, as already described for *tet* genes (Rhodes et al., 2000; Schmidt et al., 2001a). Some *qnr* genes have also been described in the Vibrionaceae family and it is suggested that water-borne Vibrionaceae may constitute a natural reservoir for Qnr-like quinolone-resistance determinants (Poirel et al., 2005; Cattoir et al., 2007; Cattoir and Nordmann, 2009). Evidence suggests that *qnr*-plasmids are most commonly integron associated and carry multiple resistance determinants, providing resistance to several classes of antimicrobials, including beta-lactams and aminoglycosides (Li, 2005).

### TETRACYCLINE RESISTANCE

Oxytetracycline is a broad-spectrum bacteriostatic antimicrobial, active against a wide variety of Gram-positive and Gram-negative bacteria, which is extensively used in fish farming. Tetracyclines bind reversibly to the 70S ribosome of prokaryotes and block protein synthesis (Chopra, 1985; Roberts, 1996).

Mechanisms of tetracycline resistance include active efflux, ribosomal protection, ribosomal RNA mutations, and tetracycline inactivation (Speer and Salyers, 1989; Salyers et al., 1990; Burdett, 1991; Levy, 1992; Speer et al., 1992; Taylor and Chau, 1996). Tetracycline resistance in fish farm-associated bacteria has been found to be mainly mediated by one or more of the Tet family of proton-dependent efflux pumps or via ribosomal protection by cytoplasmic proteins found widely in Gram-negative bacteria (Roberts, 2005; Roberts et al., 2012).

Several *tet* determinants have been identified in fish farm bacteria from a number of geographical locations and fish species (DePaola et al., 1988; Adams et al., 1998; Rhodes et al., 2000; Schmidt et al., 2001a; Furushita et al., 2003; Miranda et al., 2003; Akinbowale et al., 2007; Seyfried et al., 2010; Gao et al., 2012). The genes *tet*(A), *tet*(B), *tet*(E), *tet*(H), *tet*(L), *tet*(34), and *tet*(35) were found in tetracycline-resistant bacteria isolated from Chilean salmon farms (Miranda et al., 2003). Recently, Seyfried et al. (2010) detected the presence of *tet*(A), *tet*(B), *tet*(D), *tet*(E), *tet*(G), *tet*(M), *tet*(O), *tet*(Q), *tet*(S), and *tet*(W) genes in medicated and non-medicated feed samples and water samples from non-commercial fish farms in the United States. Similarly, Jun et al. (2004) found a high prevalence of *tet*(A) and *tet*(D) genes associated with mobile plasmids and *tet*(B) and *tet*(G) genes associated with non-mobile elements in *Edwardsiella tarda* strains isolated from fish farms in Korea. Nonaka et al. (2007) found a high incidence of *tet*(M)-carrying *Vibrio* strains in fish farms and Agersø et al (2007) detected *tet*(E) in *Aeromonas* strains from Danish fish farms associated with large plasmids capable of horizontal transfer to *Escherichia coli*. Nawaz et al. (2009) found a high prevalence of *tet*(B) and, to a lesser extent, *tet*(A), *tet*(C) and the co-occurrence of *tet*(A) and *tet*(B) in *Escherichia coli* isolated from farm-raised catfish. Kim et al. (2004) reported the occurrence of *tet*(M) and *tet*(S) in tetracycline-resistant bacteria from fish farms in Korea and Nonaka and Suzuki (2002) found the novel OTC-resistance determinant *tet*(34) in a *Vibrio* strain isolated from cultured yellowtail (*Seriola quinqueradiata*).

The spread of *tet* genes is often facilitated by their location on mobile genetic elements, such as plasmids and transposons (Roberts, 1994; DePaola and Roberts, 1995; Chopra and Roberts, 2001). Tn1721 and Tn1721-like elements, for example, are known to play a significant role in the global dissemination of the *tet*(A) gene (Rhodes et al., 2000; Sørum, 2003; Chenia and Vietze, 2012). Miranda et al. (2003) found the *tet*(H) gene as part of the transposon Tn5706 in *Moraxella* and *Acinetobacter* strains isolated from salmon farms. In addition, they were able to transfer *tet*(B) and *tet*(34) genes from strains of *Serratia liquefaciens*, *Pseudomonas pseudoalcaligenes*, and *Brevundimonas vesicularis*. Similarly, Furushita et al. (2003) transferred *tet*(B),* tet*(D), and *tet*(Y) genes from bacterial isolates from different Japanese fish farms by conjugation and Adams et al. (1998) reported that various OTC-resistant isolates of *Aeromonas salmonicida* transferred R-plasmids carrying the *tet*(A) gene to environmental and clinical isolates of *Aeromonas* spp. Class 1 integrons harboring different combinations of the resistance gene cassettes *ant(3″)Ia, aac(6″)Ia, dhfr1, oxa2a*, and/or *pse1* and *tet* genes were also detected in a large number of plasmid bearing *Aeromonas* spp. strains isolated from tilapia, trout, and koi cultures in South Africa (Jacobs and Chenia, 2007). In *Edwardsiella ictaluri* strains isolated from diseased freshwater catfish in Vietnam, Dung et al. (2009) found the *tet*(A) gene associated with a high-molecular weight plasmid belonging to the Inc*K* group. In addition, all strains were able to transfer their *tet*(A)-carrying plasmids to *Escherichia coli* recipients.

Several studies have also reported the co-occurrence of tetracycline and sulphonamide resistances genes. Agersø (2007) showed that *tet*(39) and *sul2* genes located on plasmids of different sizes to be common among clonally distinct *Acinetobacter* spp. from fish farms in Thailand. Su et al. (2011) detected the genes *tet*(A), *tet*(C), and the sulphonamide-resistance gene, *sul2*, in more than 50% of the strains of Enterobacteriaceae they isolated from fish farms in China. Gao et al. (2012) recently reported the co-occurrence of tetracycline- and sulphonamide-resistance genes in *Bacillus* species isolated from aquaculture farms in China.

### PHENICOL RESISTANCE

Florfenicol is a synthetic fluorinated analog of chloramphenicol whose bacteriostatic activity is based on a reversible binding to the 50S subunit of 70S bacterial ribosomes that prevents peptide elongation (Schwarz et al., 2004). The replacement of a hydroxyl group with a fluorine atom protects florfenicol from inactivation by chloramphenicol acetyltransferases (CATs), a common mechanism of bacterial resistance to chloramphenicol (Shaw and Leslie, 1991; Schwarz et al., 2004). The effectiveness of florfenicol against a number of relevant fish pathogens makes it a very valuable drug for the fish farming industry (Fukui et al., 1987; Nordmo et al., 1994; Samuelsen et al., 1998, 2003; Bruun et al., 2000; Gaunt et al., 2003; McGinnis et al., 2003; Michel et al., 2003; Samuelsen and Bergh, 2004).

Mechanisms of resistance to florfenicol include specific and non-specific drug transporters, RNA methyltransferases, and specific hydrolases (Paulsen et al., 1996; Schwarz et al., 2004; Poole, 2005; Long et al., 2006; Tao et al., 2012). Genes *floR* and *fexA* belong to the major facilitator superfamily and code for efflux proteins that export florfenicol out of the cell (Schwarz et al., 2004). The gene *cfr*, which has been shown to be an RNA methyltransferase that belongs to the recently discovered radical *S*-adenosylmethionine (SAM) superfamily of proteins (Sofia et al., 2001), inhibits ribose methylation and thereby causes resistance to florfenicol, chloramphenicol, and clindamycin (Long et al., 2006).

Most studies of florfenicol resistance in fish farming have reported the occurrence of the *floR* gene. Dang et al. (2007) detected the *floR* gene in tetracycline-resistant bacteria isolated from aquaculture sites in China, and Ishida et al. (2010) detected the *floR* gene in four strains of Gram-negative bacteria isolated from fish farms in Africa. In North America, McIntosh et al. (2008) reported the occurrence of *Aeromonas salmonicida* strains carrying a conjugative IncA/C plasmid harboring *floR*, *sul2*, and *tetA* genes that were transferable to *Aeromonas hydrophila* and *Edwardsiella tarda*. Welch et al. (2009) detected IncA/C plasmid-mediated florfenicol resistance in the catfish pathogen *Edwardsiella ictaluri*, and Gordon et al. (2008) reported a multiresistant *Aeromonas bestiarum* strain carrying a plasmid harboring the *floR*, *tetY*, *sul2*, and *strA–strB* resistance genes. A relatively recent study showed that many florfenicol-resistant bacterial strains isolated from Chilean salmon farms carried the *floR* gene, whereas others possessed non-specific efflux pumps that conferred florfenicol resistance (Fernández-Alarcón et al., 2010). In fish farm impacted marine sediments, Buschmann et al. (2012) recently reported the occurrence of several strains containing plasmid-borne *floR*, *tet*, and *qnr* genes.

## PRIORITIZING RESEARCH AND POLICY NEEDS

The studies discussed in the previous sections support the hypothesis that fish farms represent a reservoir of diverse ARGs, many of which may be readily mobilized by lateral gene transfer. Given the extent of global aquaculture and its fast-paced growth, it is imperative that research needs with regards to the use of antimicrobials and the emergence and potential spread of antimicrobial resistance are prioritized. This research should form the basis of sound, science-based policies that contribute to the sustainability of the aquaculture industry and minimize risks to public health. Further studies are needed to explore the prevalence of antimicrobial resistance in zoonotic fish pathogens such as *Aeromonas hydrophila*, *Edwardsiella tarda*, *Mycobacterium fortuitum*, *Mycobacterium marinum*, *Photobacterium damselae*, *Pseudomonas fluorescens*, and *Streptococcus iniae* (Austin and Austin, 2012). Moreover, there are important knowledge gaps regarding the co-occurrence of antimicrobial-resistant bacteria from aquaculture environments with human pathogens throughout production cycles and across a range of aquaculture environments, the transfer rate of resistance genes between aquaculture and clinically relevant bacteria under field or semi-field conditions, and the epidemiology of antimicrobial resistance in areas of intense aquaculture activity.

Good management strategies can make a significant contribution to minimize the use of antimicrobials in fish and the emergence and spread of antimicrobial resistance in aquaculture environments. Among these precautionary practices are the use of “good quality” fish stocks, reducing stocking densities, maintaining overall good environmental conditions (e.g., dissolved O_2_ levels), the implementation of proper biosecurity measures, the development of effective vaccines and vaccination programs, and the rotation of antimicrobial compounds in the treatment of fish disease. Although the use of antimicrobials in human medicine places constraints on the type of antimicrobials that may be used in veterinary medicine, the rotation of antimicrobials in aquaculture may play an important role in reducing the chances of selection, co-selection, and dissemination of antimicrobial resistance. In this context, it is also important that efforts are directed toward creating incentives for the development and registration of antimicrobials for aquaculture use in addition to implementation of antimicrobial stewardship practices.

## Conflict of Interest Statement

The authors declare that the research was conducted in the absence of any commercial or financial relationships that could be construed as a potential conflict of interest.
